# Self-Evisceration of Intestines as the Initial Presentation of Schizoaffective Disorder

**DOI:** 10.1155/2023/4334552

**Published:** 2023-03-13

**Authors:** Stephanie Hamlin, Dana L. Sharma, Anita S. Kablinger

**Affiliations:** ^1^Virginia Tech Carilion School of Medicine, Roanoke, VA, USA; ^2^Department of Psychiatry and Behavioral Science, Carilion Clinic, Roanoke, VA, USA

## Abstract

Schizoaffective disorder is categorized by major mood episodes and symptoms of schizophrenia that include disorganized speech, delusions, paranoia, and hallucinations. It is associated with risk factors, including a history of abuse and cannabis use, and patients are typically diagnosed in adolescence and young adulthood. In this case report, we describe the unusual case of a 39-year-old male patient with undiagnosed schizoaffective disorder who self-eviscerated his intestines during an episode of psychosis. He received an emergent exploratory laparotomy with a partial colectomy. After medical stabilization and reorientation, the patient recalled a 10-year history of paranoia associated with significant cannabis use, despite otherwise functioning appropriately in society. During a two-week hospital course, his paranoia and hallucinations were remitted on olanzapine and valproic acid. In addition to discussing his presentation and recollection of the incident, we also discuss similar cases of self-mutilation in nonsuicidal patients and the relationship between cannabis use and schizophrenia spectrum disorders.

## 1. Introduction

Schizoaffective disorder is categorized by major mood episodes with symptoms of schizophrenia that include disorganized speech, delusions, paranoia, and hallucinations. The incidence of self-harm is high among this disorder, as with patients with schizophrenia spectrum disorders. This diagnosis has a lifetime prevalence of around 0.3% [1]. Males are usually diagnosed earlier than females, with a peak age of onset between 21 and 25 years old [2]. Although the exact mechanism underlying the pathophysiology of schizoaffective disorder is unclear, several neurotransmitters are implicated, including dopamine, serotonin, and norepinephrine. Further, white matter abnormalities in several brain regions, including the right precuneus, left temporal gyrus, and the right lentiform nucleus, are associated with schizoaffective disorder. Additionally, chronic cannabis use can contribute to the development of psychotic symptoms in specific patient populations via mechanisms that modulate dopamine transmission [3]. The association between cannabis and psychosis has become of increased interest with the widespread legalization of recreational and medicinal cannabis in the past decade in the United States. Treatment for schizoaffective disorder is tailored to the individual with the mainstay being antipsychotic pharmacotherapy with adjunctive mood stabilizers and antidepressants when indicated. Patients should also receive psychotherapy, whether it is individual or group. Electroconvulsive therapy (ECT) is also indicated in treatment-resistant cases, especially when symptoms of catatonia or aggression are present [1]. Here, we describe an unusual case of self-mutilation in a patient with chronic cannabis abuse who presents later in life with undiagnosed schizoaffective disorder and is ultimately stabilized on scheduled olanzapine and valproic acid.

## 2. Case Report

A 39-year-old Caucasian male presented to inpatient psychiatry after initially presenting to the emergency department with psychosis. He had been found crawling in a parking lot with several feet of intestines dragging on the ground. The patient was only responding internally during the examination, and a urine drug screen was positive only for cannabis. He received an emergent exploratory laparotomy with a partial colectomy. He stabilized medically but became briefly catatonic on day six of hospitalization, which was resolved with intravenous diazepam. On day seven, he was transferred to the inpatient psychiatry unit on a voluntary basis.

On admission to inpatient psychiatry, the patient was fully oriented and able to recall stabbing himself in the abdomen because characters in his mind told him to, having an out-of-body experience as he watched himself pull out several feet of intestines. He felt compelled to follow the voices' commands because he had guilt about his history of sexual abuse towards women. He then recalls running out of his apartment and waking up in the hospital. He endorsed paranoia and auditory hallucinations for the last 10 years but never sought treatment. He felt hopeless, yet believed his potential was infinite. He had no suicidal ideation and expressed a fear of hurting himself upon returning home if the voices persisted.

He had never been diagnosed with a psychiatric illness or received treatment, but he did see a psychiatrist at age 17 after he was charged with indecent exposure. He recalled periods in his life when he had little need for sleep, had racing thoughts, and felt that signs and advertisements were speaking directly to him. His social history was notable for heavy cannabis use beginning at age 18. He was a daily user for 5-6 years and began experiencing paranoia and auditory hallucinations. Despite quitting for two years, the psychotic symptoms persisted, and he reported currently using cannabis 3-4 days per week. He also had a history of trauma and was emotionally and physically abused by his father who had alcohol use disorder.

On a physical exam, the patient had normal speech and appearance, an expansive affect, and poor insight. He endorsed auditory hallucinations of male voices that were commanding and feelings of paranoia. Given this patient's history of mood fluctuation and psychosis for years, even in the absence of cannabis use, he was diagnosed with schizoaffective disorder, bipolar type. He responded to olanzapine 10 mg twice daily and valproic acid 500 mg three times daily, and he was discharged after two weeks of hospitalization.

## 3. Discussion

To our knowledge, this is the third report of self-evisceration of the intestines as the initial presentation of a schizophrenia spectrum disorder in a nonsuicidal patient. A 2015 case report described a patient who gave himself a laparotomy incision and cut out his transverse colon with the rationale of wanting to explore his abdomen. The patient had undiagnosed schizophrenia and was psychotic but not suicidal [4]. A 2013 report described a similar case where a patient self-eviscerated his intestines due to commanding auditory hallucinations. This patient had no psychiatric history but was found to have schizophrenia which was exacerbated by alcohol intoxication at the time of self-injury [5]. In each case, patients' remaining intestines were anastomosed around the defect. A colostomy was avoided to prevent the risk of further manipulation of the intestines by the patient and to avoid barriers to inpatient rehabilitation. There are numerous other reported cases of self-evisceration of the intestines as suicide attempts in patients with psychiatric illness. This patient experienced psychosis for a decade prior to self-evisceration, and this case serves to emphasize the importance of risk factor identification and intervention in patients with suspected psychosis to avoid significant morbidity and disease burden on the healthcare system.

This patient had several risk factors for developing a schizophrenia spectrum disorder. He described a history of emotional and physical trauma throughout his childhood after his mom died when he was two years old. In a meta-analysis of patients with schizophrenia, the incidence of childhood physical and emotional trauma was reported to be between 38% and 50% of patients [6]. He also had a significant history of cannabis use for over 20 years. It is well known that cannabis use can contribute to psychotic symptoms. Studies in recent years have demonstrated that daily use of marijuana increases the risk of psychotic illness development up to five times [3]. Although this exact mechanism is still under research, it has been found that chronic marijuana users who have a specific variant of the AKT1 gene are at an increased risk of developing psychosis. This gene codes for an enzyme that affects dopamine signaling in the striatum. An increased risk of psychosis was also seen in adults who used cannabis in their teen years and possessed a specific variant of the catechol-O-methyltransferase (COMT) gene, which codes for an enzyme involved in dopamine and norepinephrine degradation [7]. With the recent widespread legalization of cannabis for both recreational and medical use in states across the nation, further research into the pathophysiology of how cannabis contributes to psychosis is essential. Understanding this mechanism would allow for improved risk stratification and potentially the future development of screening tests for at-risk individuals.

Future directions may include further investigating the relationship between cannabis use disorder and schizophrenia. There are conflicting hypotheses that state patients with schizophrenia are more likely to use cannabis and that cannabis use may precipitate schizophrenia. Larger-scale and retrospective studies support the latter hypothesis [8, 9].

This further emphasizes the need for more rigorous or prospective analyses to better inform patients with cannabis use disorders of these health risks.

## 4. Conclusion

Here, we describe a case in which a patient presented initially at age 39 with self-evisceration of the intestines and a past medical history significant for emotional/physical abuse as a youth and 20 years of chronic cannabis use. At this encounter, he was diagnosed with schizoaffective disorder and improved on olanzapine and valproic acid. Several factors contributed to his presentation including a history of trauma increasing his risk for schizophrenia spectrum disorders and heavy cannabis use that may have exacerbated his psychosis. The relationship between psychosis and cannabis has been a major topic in recent years with the widespread legalization of recreational cannabis consumption across the United States. Although recent research has demonstrated potential gene variations including AKT1 and COMT, which may predispose individuals to the development of psychotic symptoms, further research is necessary to delineate the exact mechanism. If specific gene variations or biomarkers can be defined and have predictive value, screening tests could be developed to allow physicians to provide better risk stratification to this subgroup of patients, which may help reduce morbidity, mortality, and disease burden on the healthcare system.
